# Phase Behavior and Its Effects on Crystallization in a Poly(trimethylene terephthalate)/Phenoxy Resin Blend

**DOI:** 10.3390/polym8010021

**Published:** 2016-01-19

**Authors:** Su Jung Cho, Jae Han Cho, Kwang Hee Lee

**Affiliations:** Department of Polymer Science and Engineering, Inha University, Incheon 22212, Korea; 22142074@inha.edu (S.J.C.); 22151217@inha.edu (J.H.C.)

**Keywords:** poly(trimethylene terephthalate), phenoxy resin, phase behavior, crystallization

## Abstract

Phase behavior and its effects on crystallization in an extruded poly(trimethylene terephthalate) (PTT)/phenoxy resin blend were studied with time-resolved light scattering (TRLS), optical microscopy (OM), differential scanning calorimetry (DSC), and small-angle X-ray scattering (SAXS). During annealing in the molten state, a two-phase structure with unique periodicity and phase connectivity was developed by liquid–liquid phase separation. After the formation of the phase-separated structure, the blend was homogenized by the interchange reactions between the two polymers. The crystallization behavior of PTT predominantly depended on the phase morphology developed during annealing. The pre-existing phase structures disturbed the lamellar orientation, resulting in a poorly ordered spherulitic superstructure.

## 1. Introduction

It has been reported that the morphology of crystalline polymer blends is sometimes influenced by a liquid-liquid (L–L) demixing process [1,2,3,4,5,6]. If the crystalline polymer blend has a phase diagram, crystallization may take place simultaneously and compete with L–L phase separation [7]. The two competitive processes may create unique morphological patterns that are not attainable by either process alone.

Blends of poly(trimethylene terephthalate) (PTT) and phenoxy resin are basically immiscible over a wide range of compositions. During melt processing, interchange reactions between the phenoxy hydroxyl and ester type carbonyl groups lead to the formation of copolymers, enhancing the miscibility of the blends [8,9,10,11]. With an increase in the reaction extent, the miscibility of the blends changes from complete incompatibility to partial compatibility and finally to complete compatibility. PTT/phenoxy resin blends thus provide a unique opportunity for investigating the effects of various liquid-phase changes on crystalline morphology.

In this work, time-resolved light scattering (TRLS) and optical microscopy (OM) observations were performed to study the phase behavior in an extruded PTT/phenoxy resin blend. The crystallization and the melting behavior were examined using differential scanning calorimetry (DSC). The characteristics of the crystalline morphology were discussed on the basis of the effects of combined crystallization and L–L demixing.

## 2. Experimental

### 2.1. Materials

Commercial PTT (*M*_W_ = 23,000, *M*_W_/*M*_n_ = 2.1) produced by SKC Co. (Seoul, Korea) was used. The phenoxy resin (*M*_W_ = 52,000, *M*_W_/*M*_n_ = 4.0) was supplied by Union Carbide Co. (Houston, TX, USA). It is worth mentioning that the effect of molecular weight and molecular weight dispersity is of particular interest because some variations of these parameters can cause significant changes in crystallization behavior [12]. However, the influence of molecular weight and molecular weight dispersity was not considered in this work. After being dried in a vacuum oven at 120 °C for 24 h, PTT and phenoxy resin were melt-mixed at 265 °C on a 19 mm co-rotating twin-screw extruder (BA-19, Bautek, Pocheon, Korea) at 200 rpm. The extrudate was quenched in ice water to freeze the structure in the molten state and then chopped into pellets. The composition of the blend was 50/50 by weight.

### 2.2. TRLS and OM

A thin-film specimen (*ca.* 15 μm thick) was prepared by pressing the blend pellets between two cover glasses at 265 °C. Immediately after melt-pressing, the specimen was quickly transferred onto a hot stage of a light scattering photometer equipped with a charge-coupled device camera, and the angular distribution of the light scattering intensity was detected. The sample was held at 265 °C for a designated time (*t*_s_), and then rapidly transferred onto a second hot stage set at a desired temperature to examine the crystallization. A polarized He–Ne gas laser with 632.8 nm wavelength was applied to the film specimen. Two optical geometries were employed: *V*_v_ geometry, where the optical axis of the analyzer is set parallel to that of the polarizer, and *H*_v_ geometry, where the two axes are arranged perpendicularly. The phase morphology and the spherulitic texture were also observed with OM (Nikon Optiphot 2, Tokyo, Japan).

### 2.3. Differential Scanning Calorimetry (DSC)

The crystallization and the melting behavior were investigated with a Perkin-Elmer DSC-7 differential scanning calorimeter (Waltham, MA, USA). The extruded sample was first melted at 265 °C for *t*_s_ and then rapidly cooled to the desired crystallization temperature. After crystallizing isothermally for 1 h, the sample was heated to 300 °C at a rate of 10 °C/min in a nitrogen atmosphere.

### 2.4. Small-Angle X-ray Scattering (SAXS) 

SAXS measurements were performed on beam line 4C1 at the Pohang Light Source, Pohang, Korea. The storage ring was operated at an energy level of 2 GeV. The SAXS equipment employs point focusing optics with a Si double crystal monochromator followed by a bent cylindrical mirror. The incident beam intensity, with a wavelength of 0.149 nm, was monitored by an ionization chamber for correction of any minor decrease of the primary beam intensity during the measurement. The scattering intensity by thermal fluctuations was subtracted from the SAXS profile *I(q)* by evaluation of the slope of a plot of *I(q)·q^4^ vs.* *q^4^* at a wide scattering vector *q*, where *q* is (4π/λ)sin(θ/2), λ and θ being the wavelength and scattering angle, respectively [13].

## 3. Results and Discussion

**Figure 1:**
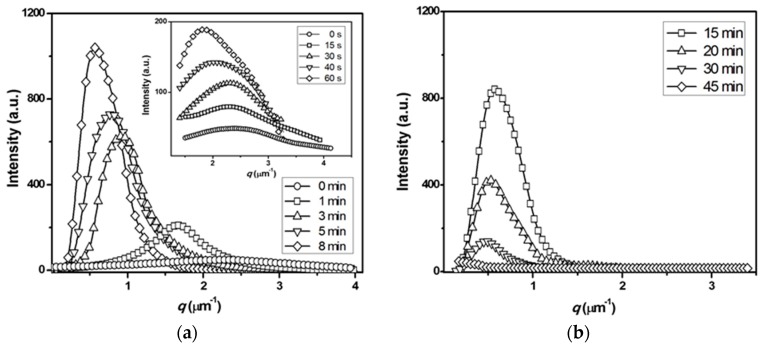
Change in the one-dimensional *V*_v_ light scattering profiles of the PTT/phenoxy resin blend during annealing at 265 °C: (**a**) early stage of annealing; (**b**) later stage of annealing. The insert of this figure shows the *V*_v_ profiles in the initial stage (*t*_s_ ≤ 60 s) of annealing.

Figure 1 shows the changes in the one-dimensional *V*_v_ scattering profiles. Even at *t*_s_ = 0 min, a weak scattering peak appears at large *q*, suggesting an appreciable development of L–L phase separation in the quenched and remelted specimen. In the early stage of *t*_s_ ≤ 30 s, the peak intensity *I*_m_ increases with *t*_s_, but its position *q*_m_ remains constant. *I*_m_ subsequently increases with *t*_s_, and *q*_m_ shifts to smaller angles, implying that domain growth takes place with some regularity. From the scattering results, it appears that the L–L phase separation proceeds by demixing via spinodal decomposition (SD). In the later stage of annealing, *q*_m_ further shifts toward smaller angles in the beamstop area, and the scattering intensity decreases with *t*_s_. At *t*_s_ = 45 min, the intensity becomes very weak and shows almost no *q*-dependence, suggesting that phase homogenization is nearly obtained in the blend. This scattering feature due to L–L phase separation and subsequent homogenization during annealing is consistent with results obtained for other systems such as poly(ethylene terephthalate) (PET)/poly(ethylene-2,6-naphthalate) (PEN), PET/polycarbonate (PC), and PET/phenoxy resin blends [14,15].

**Figure 2:**
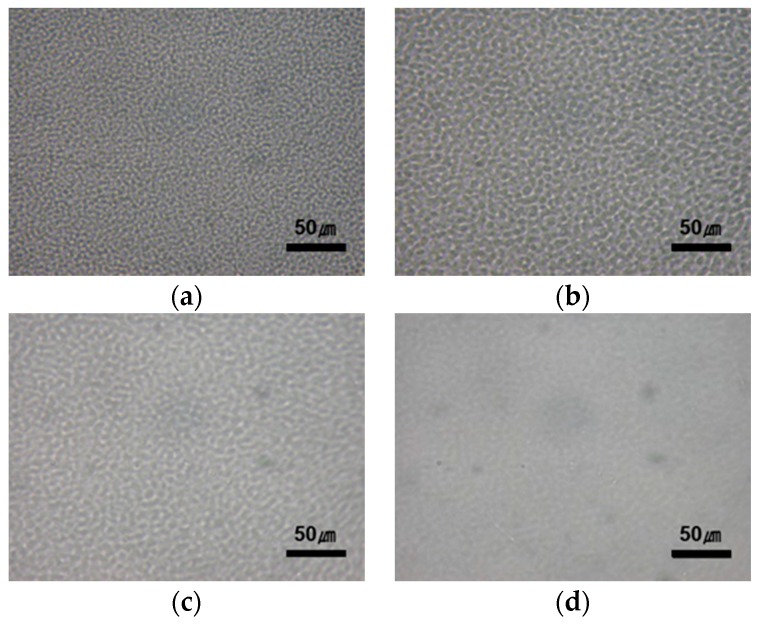
Optical micrographs of the PTT/phenoxy resin blend after annealing at 265 °C for (**a**) *t*_s_ = 0.5 min; (**b**) *t*_s_ = 8 min; (**c**) *t*_s_ = 15 min; (**d**) *t*_s_ = 45 min.

Supplemental evidence of L–L phase separation and homogenization was provided through OM observations. Figure 2 shows optical micrographs of the PTT/phenoxy resin blend annealed at 265 °C for *t*_s_. In the initial stage (Figure 2a), interconnectivity in both phases can be seen and the phases are regularly spaced. A two-phase structure with unique periodicity and phase connectivity is one of the hallmarks of SD. At the later stage of L–L phase separation (Figure 2b), the space of the phase connectivity increases. As the interchange reactions between the two polymers proceed, the domain growth is suppressed and the blend morphology is mainly controlled by the phase homogenization process (Figure 2c). Finally, the blend shows a homogeneous mixture (Figure 2d).

**Figure 3:**
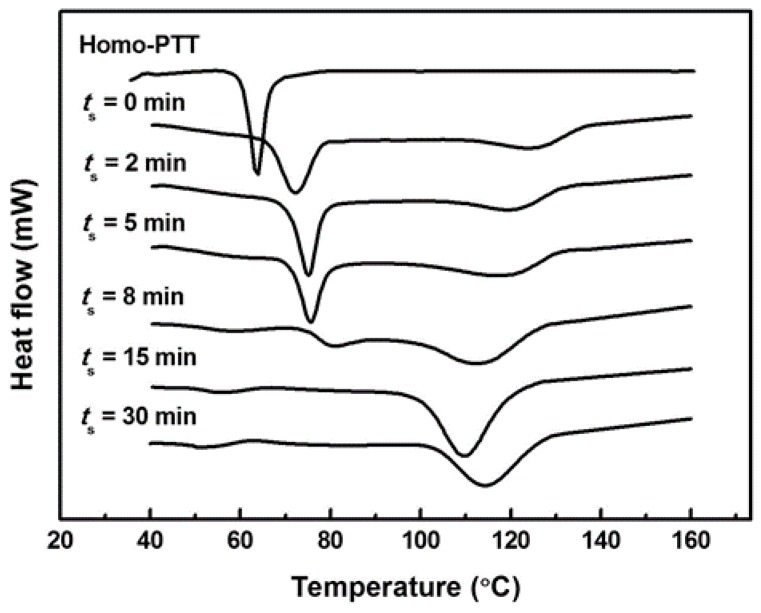
DSC heating thermograms of homo-PTT and blend samples. Homo-PTT was quenched after annealing at 265 °C for 5 min and blend samples were quenched after annealing at 265 °C for *t*_s_.

The crystallization behavior of the PTT/phenoxy resin blend appears to be significantly different from that of the homo-PTT, because L–L demixing and subsequent phase homogenization in the molten state cause changes in the concentration fluctuation and the chain periodicity. Figure 3 shows the cold crystallization behavior of the blend samples quenched after annealing at 265 °C for *t*_s_. Only one exotherm can be observed for the homo-PTT, whereas two exothermic peaks are identified for the blend samples. The crystallization behavior of the blends is dependent on the ability of the component to crystallize. For example, the crystallization behavior characteristic of a single mode can be expected if all the PTT molecules have the same status. However, multimode crystallization will appear if some PTT molecules have a different status. It is likely that the two different PTT portions will show different crystallization behavior, which would account for the observation of two crystallization exotherms. Therefore, the first exotherm might be due to the crystallization of PTT in the PTT-rich phase, and the second exotherm might be associated with the crystallization of PTT in the phenoxy resin-rich phase. It is worth noting that the L–L phase separation by SD is realized by up-hill diffusion; *A* molecules diffuse into an *A*-rich phase from a *B*-rich phase. Thus, in the early stage of annealing, the phenoxy resin molecules in the PTT-rich phase may be forced to migrate to the phenoxy resin-rich phase. As a result, the amount of phenoxy resin in the PTT-rich phase should be decreased with *t*_s_, and the *T*_g_ of the PTT-rich phase may correspondingly decrease. Because of the smaller degree of impurities and the lower *T*_g_ in the PTT-rich phase, the crystallization rate of PTT in the PTT-rich phase for the samples quenched from the early-to-intermediate stages of SD is expected to be faster than that for the sample of *t*_s_ = 0 min. However, it is observed that the cold crystallization peaks of PTT in the PTT-rich phase shift to higher temperatures with increasing *t_s_*, and finally combine with those in the phenoxy resin–rich phase. This result suggests that the crystallization of the PTT constituent is predominantly influenced by the disruption of chain periodicity due to the interchange reactions rather than the composition change between separated phases.

**Figure 4:**
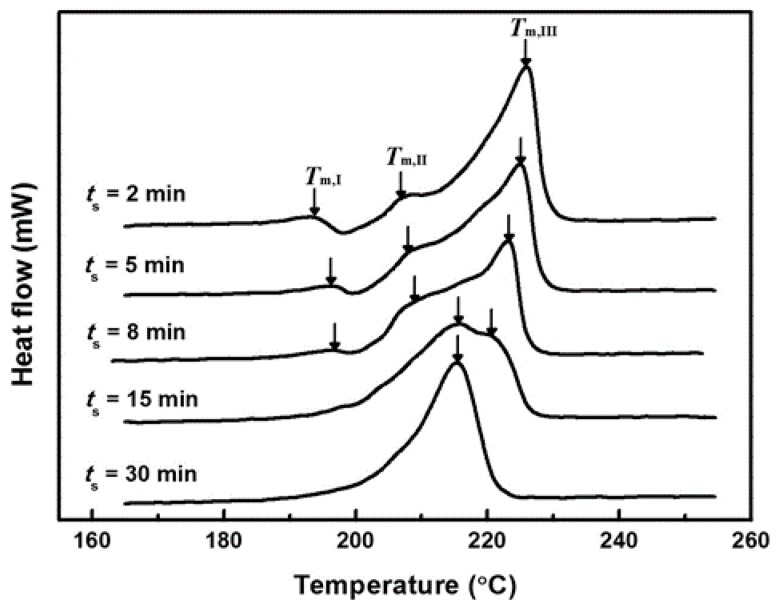
DSC heating thermograms of blend samples crystallized isothermally at 155 °C for 1 h after annealing at 265 °C for *t*_s_.

Figure 4 shows DSC thermograms of blend samples crystallized isothermally at 155 °C for 1 h after annealing at 265 °C for *t*_s_. The crystallization temperature of 155 °C was chosen in order to induce the rapid growth rate of the PTT crystals [16,17]. Conceptually, it seems plausible that the rapid crystallization of PTT at 155 °C would be effective to lock-in further growth of the L–L phase separation. The samples exhibit multiple-melting endotherms. Various models have been proposed to explain the multiple-melting behavior. One of the most recent studies on this was reported by Al Raheil [18]. He used a combination of the melting-recrystallization model and the dual lamellar population model to explain the triple melting behavior. The three melting endotherms were denoted as peaks I, II, and III in order of melting point. The lowest melting peak (*T*_m,I_) was attributed to the melting of crystals formed by a secondary crystallization. The second melting peak (*T*_m,II_) was associated with the melting of crystals formed by a primary crystallization. The third melting peak (*T*_m,III_) was assigned to the melting of crystals formed by a reorganization process during the DSC scan. One interesting feature in the figure is that the peak area of *T*_m,II_ increases with *t*_s_, whereas the *T*_m,III_ peak decreases and finally disappears. In the pure PTT, melting and subsequent recrystallization of primary crystals occur simultaneously, and as a result the two melting peaks, *T*_m,II_ and *T*_m,III_, merge (data not shown). However, in the blend samples, the recrystallization rate is gradually retarded during the DSC scan due to the decrease in the chain regularity of PTT components with *t*_s_. This leads to division of the two independent peaks, *T*_m,II_ and *T*_m,III_. For the samples with a prolonged annealing time (*t*_s_ ≥ 15 min), however, recrystallization is largely restricted and, hence, the peak of *T*_m,III_ appears as a shoulder or disappears in the DSC trace.

**Figure 5:**
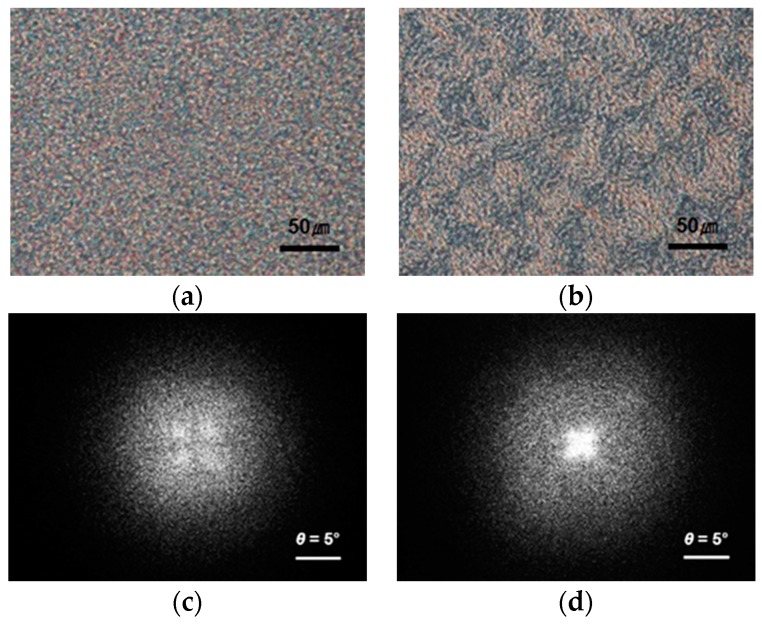
Polarized optical micrographs and the corresponding *H*_v_ light scattering patterns of blend samples crystallized isothermally at 155 °C for 1 h after annealing at 265 °C for (**a**) and (**c**) *t*_s_ = 8 min; (**b**) and (**d**) *t*_s_ = 30 min.

Figure 5 shows polarized optical micrographs and the corresponding small-angle light scattering (SALS) *H*_v_ patterns for blend samples annealed for *t*_s_ = 8 and 30 min and subsequently crystallized isothermally at 155 °C for 1 h. No samples show spherulitic texture, which is similar in appearance to a “Maltese Cross” extinction pattern. This indicates that the pre-existing phase structures disturb the lamellar orientation, resulting in a poorly ordered morphology. It is worth noting that the SALS patterns provide direct evidence of the evolution of a spherulitic superstructure, even though the formation of spherulites is not observed optically. The sample of *t*_s_ = 8 min shows a diffuse and less azimuthally dependent scattering pattern with lobs at 45° to the polarization direction. The *H*_v_ scattering pattern is related to the large-scale arrangement of the individual lamellar crystallites. Stein and Chu [19] reported that lower orders of organization result in a broad *H*_v_ scattering pattern, *i.e.*, as the disorder of lamellar orientation increases, the azimuthal dependence of the scattering pattern is reduced and the scattering pattern shows a diffuse pattern. The crystallization of PTT may be significantly influenced by the phase morphology developed during annealing. As the crystal growth front advances by finding and following the PTT-rich domains in the phase-separated structure, the lamellar orientation is dependent on the contour of the PTT-rich domains. Therefore, the diffuse pattern strongly suggests that the crystal growth path is highly distorted by the pre-existing phase morphology.

**Figure 6:**
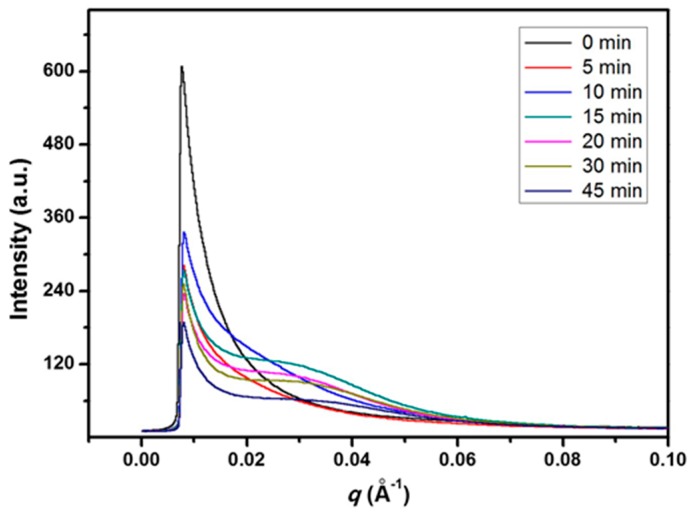
SAXS profiles of blend samples crystallized isothermally at 155 °C for 1 h after annealing at 265 °C for *t*_s_.

The SAXS profiles of the blend samples crystallized isothermally at 155 °C for 1 h after annealing at 265 °C for *t*_s_ are shown in Figure 6. The samples annealed for longer time show a broad scattering peak near *q*~0.03 Å^−1^. However, the samples annealed for *t*_s_ ≤ 10 min exhibit only scattering decay. The absence of an appreciable scattering peak for the samples of *t*_s_ ≤ 10 min implies that the lamellar morphology is significantly influenced by the phase structure developed during the early stage of annealing.

## 4. Conclusions

In this study, the phase changes and subsequent crystallization behavior in an extruded PTT/phenoxy resin blend were examined. During annealing in the molten state, the L–L phase separation proceeded. After the formation of a domain structure, the blend underwent phase homogenization by the interchange reactions between the two polymers. The thermal analysis suggested that the crystallization and the melting behavior of PTT components predominantly depended on the change of the sequence distribution in the polymer chains, which is determined by the level of the interchange reactions rather than the composition change of the separated phases. The SAXS profiles indicated that the lamellar morphology was significantly influenced by the phase structure developed during the early stage of annealing.
